# Temporobasales Meningeom als mögliche Ursache einer Kognitionsstörung bei Depression im höheren Lebensalter

**DOI:** 10.1007/s00115-024-01787-2

**Published:** 2024-12-20

**Authors:** Meret Heibel, Horst Urbach, Katharina Domschke, Sabine Hellwig

**Affiliations:** 1 Klinik für Psychiatrie und Psychotherapie, Universitätsklinikum Freiburg, Medizinische Fakultät, Albert-Ludwigs-Universität Freiburg, Hauptstr. 5, 79104 Freiburg, Deutschland; 2 Klinik für Neuroradiologie, Universitätsklinikum Freiburg, Medizinische Fakultät, Albert-Ludwigs-Universität Freiburg, Freiburg, Deutschland

## Anamnese

Eine 83-jährige, agile und selbstversorgende Patientin (Rechtshänderin) stellte sich zur stationär-psychiatrischen Behandlung bei anhaltender depressiver Symptomatik mit im Vordergrund stehenden kognitiven Defiziten vor. Bis zwei Wochen vor Aufnahme war bereits eine stationäre Behandlung in einer anderen Klinik erfolgt, wobei trotz medikamentöser Kombinationsbehandlung mittels Clompiramin und Lithium nur eine Teilremission der depressiven Symptomatik erreicht werden konnte. Eine rezidivierende depressive Störung bestand seit den 1970er-Jahren, welche mehrere stationäre Vorbehandlungen und diverse medikamentöse Therapieversuche notwendig gemacht hatte. Die Einweisungsdiagnose der ambulanten psychiatrischen Behandlerin lautete: schwere depressive Episode bei rezidivierender Störung mit Pseudodemenz.

## Psychischer Befund

Im Aufnahmegespräch imponierten ein depressiver Affekt, ein reduzierter Antrieb mit gesteigerter Ermüdbarkeit, Interessen- und Freudverlust sowie ein reduziertes Selbstwertempfinden. Die Patientin berichtete auch von passiven Todesgedanken, distanzierte sich aber klar von einer akuten Suizidalität. Korrespondierend fand sich im Beck-Depressions-Inventar (BDI-II) mit 26 Punkten eine mittelschwere depressive Symptomatik. Des Weiteren beklagte die Patientin Aufmerksamkeits- und Konzentrationsstörungen und eine Beeinträchtigung ihrer Gedächtnisleistungen. In der klinischen Prüfung fanden sich keine Auffassungsstörungen, jedoch leicht- bis mittelgradige Aufmerksamkeits- und Konzentrationsstörungen. Im Mini-Mental-Status-Test (MMST) ließ sich mit 26 von maximal 30 Punkten ein leichtes kognitives Defizit objektivieren. Die episodischen Gedächtnisleistungen als links hippokampale Funktion waren beeinträchtigt, wobei null von drei Wörtern im Spätabruf erinnert wurden. Zudem war die Rückwärtssubtraktion („100 minus 7“) eingeschränkt möglich.

## Somatischer Befund

Die körperliche Untersuchung war sowohl internistisch als auch neurologisch unauffällig.

## Diagnostik, Therapie und Verlauf

Im Routinelabor fanden sich bis auf eine Vitamin-B12-Mangelanämie, die substituiert wurde, keine weiteren Auffälligkeiten. Der Clomipraminserumspiegel lag unter einer Tagesdosis von 75 mg mit 280 µg/l im unteren Normbereich (Norm: 230–450 µg/l). Der Lithiumserumspiegel betrug 0,43 mmol/l bei einer Tagesdosis von 450 mg, welches unter Berücksichtigung des höheren Lebensalters als suffiziente Augmentationstherapie eingestuft wurde. Ein EEG blieb ohne pathologischen Befund, insbesondere ohne Hinweis auf einen Herdbefund oder epilepsietypische Potenziale.

Als Ursache der mnestischen Defizite, welche von der Patientin als besonders beeinträchtigend und quälend wahrgenommen wurden, die jedoch testpsychologisch allenfalls in leichtgradiger Ausprägung objektivierbar waren, erwogen wir zunächst differenzialdiagnostisch eine depressionsbedingte Pseudodemenz wie auch Nebenwirkungen der potenziell inadäquaten Pharmakotherapie im Alter mit einem Trizyklikum [1]. Unter Fortführung der Lithiumaugmentation stellten wir daher die antidepressive Medikation von Clomipramin auf Venlafaxin um und dosierten dieses unter Spiegelkontrollen bis auf 112,5 mg/Tag (Summenspiegel 164,4 µg/l; Norm: 100–400 µg/l) auf. Hierunter remittierte das depressive Syndrom vollständig, was gegen das Vorliegen einer organischen affektiven Störung sprach, die kognitiven Defizite persistierten jedoch unverändert.

**Abb. 1:**
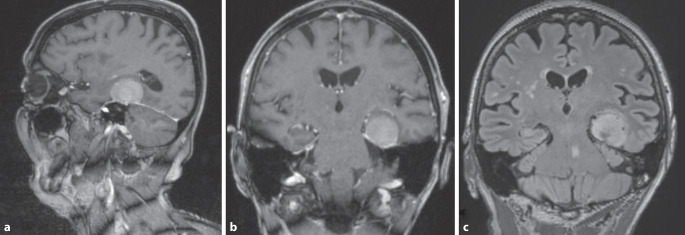
Magnetresonanztomographie des Kopfes: **a** sagittale, **b** koronar reformatierte MP-RAGE-Sequenz nach Kontrastmittelgabe, **c** koronar reformatierte FLAIR-Sequenz. Großes links temporobasales Meningeom mit Verlagerung und Kompression des linken Hippokampus

Zur weiteren Abklärung der leichten kognitiven Störung ergänzten wir daraufhin leitliniengemäß eine Magnetresonanztomographie (MRT) des Neurokraniums. Hier zeigte sich eine kontrastmittelaffine, breitflächig der Dura bzw. dem Tentoriumrand aufsitzende 29 × 25 mm große Raumforderung, a. e. einem temporobasalen Meningeom entsprechend (Abb. 1). Aufgrund der lokal raumfordernden Wirkung des Tumors mit Kompression der temporomesialen Strukturen einschließlich des Hippokampus links, werteten wir diese Läsion als ursächlich für die mnestischen Defizite und überwiesen die Patientin an die Schädelbasissprechstunde der hiesigen Klinik für Neurochirurgie zur Klärung möglicher Interventionen. In einer Verlaufs-MRT nach 6 Monaten fand sich ein stabiler Befund ohne Größenprogress des Tumors, sodass sich die Patientin gegen eine von den Kollegen primär empfohlene operative Resektion des Meningeoms entschied.

**Abb. 2:**
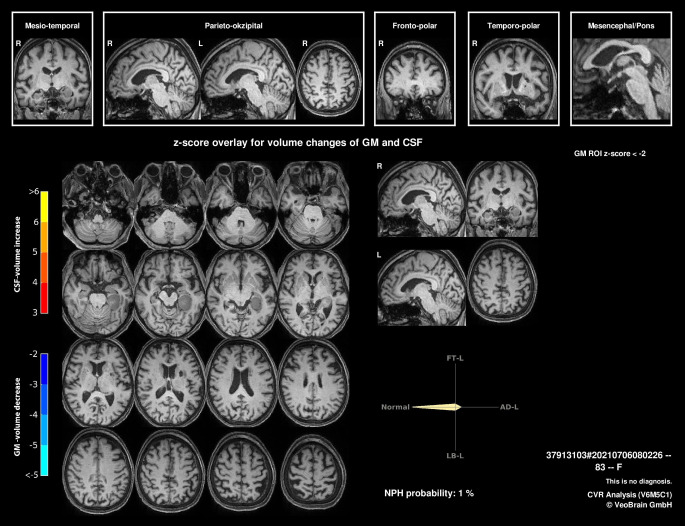
Eine T1-gewichtete MPRAGE-Sequenz einschließlich einer regionen- und voxelbasierten Analyse [9] zeigte kein spezifisches Atrophiemuster, was eine neurodegenerative Erkrankung, wie z. B. eine Alzheimer-Erkrankung, sehr unwahrscheinlich macht

Differenzialdiagnostisch war bereits initial eine neurodegenerative Ursache des amnestischen Syndroms erwogen worden, nicht zuletzt auch wegen der Vulnerabilität gegenüber Clomipramin, die ein vorbestehendes cholinerges Defizit als Frühzeichen einer möglichen Alzheimer-Erkrankung suggerierte. Die ergänzend durchgeführte MR-tomographische volumetrische Analyse ergab hierfür jedoch keinerlei Anhalt (Abb. 2). Gegenüber der darüber hinaus empfohlenen Liquordiagnostik zur Bestimmung demenzspezifischer Biomarker wie auch weiterführenden PET-Untersuchungen zeigte sich die Patientin ablehnend.

Die depressive Symptomatik zeigte sich im Längsverlauf unter Fortführung der oben genannten Medikation über mehrere Jahre vollständig remittiert, die Patientin schilderte jedoch unverändert Gedächtnisstörungen. Nachdem mit dem Befund eines Meningeoms ein möglicherweise zugrunde liegendes Korrelat geklärt, wenngleich nicht behoben werden konnte, gelang es ihr allerdings, diese Defizite besser einzuordnen und dafür eine gewisse Akzeptanz aufzubringen.

## Diskussion

**Abb. 3:**
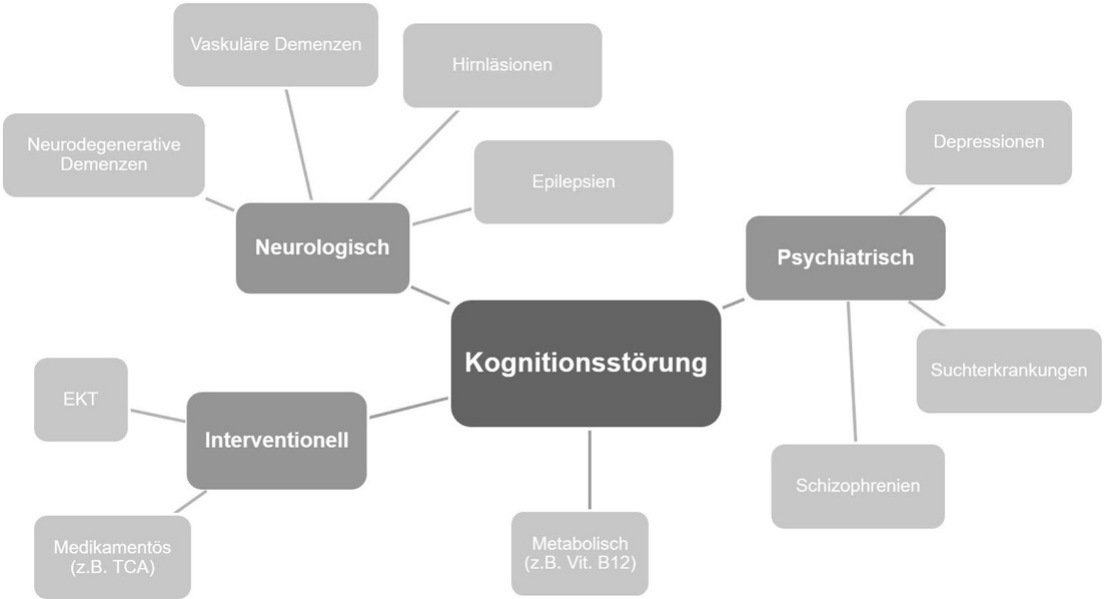
Übersicht multifaktorieller Ursachen einer kognitiven Dysfunktion bei gerontopsychiatrischen Patienten. *EKT* Elektrokonvulsionstherapie, *TCA* Trizyklika, *Vit. B12* Vitamin B12

Kognitive Defizite bei gerontopsychiatrischen Patienten sind häufig und zumeist multifaktoriell bedingt (Abb. 3), wie auch im hier geschilderten Fall. Neben einer depressiven Pseudodemenz finden sich insbesondere im höheren Lebensalter unerwünschte Effekte der Psychopharmakotherapie, so z. B. beim Einsatz trizyklischer Antidepressiva [1, 8]. Darüber hinaus gehen entzündliche ZNS-Erkrankungen wie auch neurodegenerative Demenzerkrankungen mit psychischen Symptomen einher [3, 4], wobei eine Depression sowohl Risikofaktor als auch Prodromalsymptom einer demenziellen Erkrankung sein kann [10]. Nicht zuletzt können Läsionen in strategisch relevanten Hirnregionen Ursache kognitiver Defizite sein [2].

Im hier vorliegenden Fall bleibt zu mutmaßen, dass aufgrund der Anamnese einer langjährig bestehenden rezidivierenden depressiven Störung versäumt oder darauf verzichtet wurde, in der aktuellen Indexepisode frühzeitig und konsequent nach organischen Ursachen der Symptomatik zu suchen. Die Vordiagnose hat gewissermaßen zu dieser Einschätzung verleitet und den differenzialdiagnostischen Blick „verstellt“.

Kritisch anzumerken bleibt, dass letztlich eine komorbide neurodegenerative Ursache der Kognitionsstörung nicht ausgeschlossen werden kann, da spezifische Diagnostik diesbezüglich von der Patientin initial wie auch im Verlauf nicht gewünscht wurde. Auch kann der Beweis einer kognitiven Besserung nach Resektion des Meningeoms nicht geführt werden, da die Patientin sich gegen eine Operation entschied.

## Fazit

Beim Vorliegen komorbider kognitiver Defizite bei Patienten im höheren Lebensalter (z. B. > 65 Jahre) sollte neben einer ausführlichen klinischen Befunderhebung inkl. einer neuropsychologischen Testung [6] eine Zusatzdiagnostik mittels bildgebender Verfahren wie MRT und neuronuklearmedizinischer Untersuchungen (FDG-PET, Amyloid-PET, Tau-PET), aber auch eine Liquordiagnostik inkl. der Bestimmung spezifischer Biomarker (Gesamt-tau, Phospho-tau, Amyloid-Ratio) und von Entzündungsparametern wie auch paraneoplastischer Antikörper angestrebt werden [4, 5, 7, 9].
